# Amelioration of Murine Passive Immune Thrombocytopenia by IVIg and a Therapeutic Monoclonal CD44 Antibody Does Not Require the Myd88 Signaling Pathway

**DOI:** 10.1371/journal.pone.0071882

**Published:** 2013-08-05

**Authors:** Andrew R. Crow, Honghui Yu, Dongji Han, Alan H. Lazarus

**Affiliations:** 1 The Canadian Blood Services, Toronto, Canada; 2 Department of Laboratory Medicine and the Keenan Research Centre in the Li Ka Shing Knowledge Institute of St. Michael’s Hospital, Toronto, Canada; 3 Department of Anesthesiology, Tongji Hospital, Huazhong University of Science and Technology, Wuhan, China; 4 Departments of Medicine and Laboratory Medicine & Pathobiology, University of Toronto, Toronto, Ontario, Canada; Institut National de la Santé et de la Recherche Médicale U 872, France

## Abstract

Immune thrombocytopenia (ITP) is an autoimmune bleeding disorder characterized by a low platelet count and the production of anti-platelet antibodies. The majority of ITP patients have antibodies to platelet integrin α_IIb_β_3_ (GPIIbIIIa) which can direct platelet phagocytosis by macrophages. One effective treatment for patients with ITP is intravenous immunoglobulin (IVIg) which rapidly reverses thrombocytopenia. The exact mechanism of IVIg action in human patients is unclear, although in mouse models of passive ITP, IVIg can rapidly increase platelet counts in the absence of adaptive immunity. Another antibody therapeutic that can similarly increase platelet counts independent of adaptive immunity are CD44 antibodies. Toll-like receptors (TLRs) are pattern recognition receptors which play a central role in helping direct the innate immune system. Dendritic cells, which are notable for their expression of TLRs, have been directly implicated in IVIg function as an initiator cell, while CD44 can associate with TLR2 and TLR4. We therefore questioned whether IVIg, or the therapeutic CD44 antibody KM114, mediate their ameliorative effects in a manner dependent upon normal TLR function. Here, we demonstrate that the TLR4 agonist LPS does not inhibit IVIg or KM114 amelioration of antibody-induced thrombocytopenia, and that these therapeutics do not ameliorate LPS-induced thrombocytopenia. IVIg was able to significantly ameliorate murine ITP in C3H/HeJ mice which have defective TLR4. All known murine TLRs except TLR3 utilize the Myd88 adapter protein to drive TLR signaling. Employing Myd88 deficient mice, we found that both IVIg and KM114 ameliorate murine ITP in Myd88 deficient mice to the same extent as normal mice. Thus both IVIg and anti-CD44 antibody can mediate their ameliorative effects in murine passive ITP independent of the Myd88 signaling pathway. These data help shed light on the mechanism of action of IVIg and KM114 in the amelioration of murine ITP.

## Introduction

Immune thrombocytopenia (ITP) is an autoimmune disorder characterised by the production of platelet-reactive autoantibodies which can induce platelet clearance, resulting in thrombocytopenia. Platelet clearance due to platelet integrin α_IIb_β_3_ (GPIIb/IIIa) antibodies appear to occur mainly, though not necessarily exclusively [1–4], via phagocytosis in the mononuclear phagocytic system (MPS), while antibodies to other platelet antigens may induce platelet clearance through other mechanisms [4,5].

One efficacious treatment for ITP and many other autoimmune diseases is intravenous immunoglobulin (IVIg), an IgG fraction prepared from large pools of human plasma. There are many theories as to the mechanism of action of IVIg in the treatment of autoimmune disease and the exact pathway by which IVIg functions remains incompletely understood. In the case of ITP, we have shown that IVIg increases platelet counts in mice with severe combined immune deficiency (SCID), indicating that IVIg likely mediates its effects in murine ITP at the level of the innate immune system [6,7]. One innate cell population which has been directly implicated in the IVIg pathway are dendritic cells [8], which we [9–11] and others [8,12–17] have shown can play a key role as initiators of IVIg effects.

Toll-like receptors (TLRs) are a class of pattern-recognition receptors that can play a major role in activation of the innate immune system (reviewed in 18). They are non-catalytic receptors commonly expressed on and in innate immune cells, including dendritic cells and macrophages which help allow these cells to recognize pathogen-derived macromolecules, such as bacterial/yeast cell wall components and viral/bacterial nucleic acids. Inappropriate TLR activation can disrupt immune homeostasis and potentially affect the development of inflammation and autoimmunity (reviewed in 19). Indeed, multiple murine models of autoimmunity related to defective TLR signaling exist (reviewed in 20).

All known murine TLRs except TLR3 require the adaptor protein Myd88 for TLR signaling (TLR 1, 2, 4, 5, 6, 7, 8, 9, 11, 12, 13) [21–23]. We have previously demonstrated that IVIg may form immune complexes to exert its ameliorative effects [24]. Although TLRs are classically thought of as receptors for pathogen-associated molecular patterns, it has been demonstrated that both soluble and insoluble immune complexes have the ability to engage various TLRs, and regulate inflammation [25–31].

In addition to IVIg, we have previously shown that antibodies reactive with the CD44 homing antigen can successfully ameliorate murine ITP in normal mice [32] as well as in SCID mice [7]. It has been shown that CD44 can associate with Myd88 itself [33] as well as interact with both TLR2 [34] and TLR4 [35] which both utilize Myd88. We therefore questioned whether TLR4 or the Myd88 pathway play a critical role in the ameliorative function of IVIg and a therapeutic CD44 antibody (KM114) using a murine model of ITP [6].

We demonstrate herein that acute exposure to the TLR4 ligand LPS does not inhibit the ameliorative effects of IVIg or KM114 and that IVIg was able to ameliorate murine ITP in mice with functionally inactive TLR4. In addition, both IVIg and KM114 were able to ameliorate murine ITP in Myd88 deficient mice as efficiently as in control mice. These data help define how these two therapeutics function in murine ITP.

## Materials and Methods

### Mice and reagents

Six to eight week old female control C57BL/6 mice, Myd88 deficient mice (B6.129P2(SJL)-*Myd88* ^*tm1.1Defr*^/J) and TLR4 defective C3H/HeJ mice were from Jackson Laboratories (Bar Harbor, ME). Six to eight week old female C3H/HeNCrl mice and CD1 mice were from Charles River (St-Constant, PQ). Mice were housed in micro-isolator cages and maintained on a 12 hr/12 hr light/dark cycle with food and water available *ad libitum*. Upon completion of experiments, mice were anesthetized with isoflurane and sacrificed by cervical dislocation. All animal studies were performed in accordance with the guidelines of, and under the approval of, the St. Michael's Hospital Animal Care Committee.

The IVIg used was Gamunex 10% (Telecris Biotherapeutics, Triangle Park, NC) or Privigen 10% (CSL Behring). The integrin α_IIb_ antibody (clone MWReg30) was from BD PharMingen (Mississauga, ON). The CD44 monoclonal antibody (KM114) was from BD PharMingen (Mississauga, ON) or BioXCell (West Lebanon, NH). LPS (0111:B4) was from Sigma-Aldrich (Oakville, ON). Coulter Counter diluent Isoton was from Beckman Coulter (Mississauga, ON).

### Induction and inhibition of ITP

Murine thrombocytopenia was induced by i.p. injection of 2 µg MWReg30 in 200 µL PBS, pH 7.2. For platelet counts, mice were bled via the saphenous vein. Ten µL of blood was immediately diluted in 990 µL PBS/1% EDTA, pH 7.2. The diluted blood was then centrifuged at 190xg for 2 min at room temperature to isolate platelet rich plasma (PRP). Two hundred µL PRP was then added to 10 mL Isoton diluent and platelet enumeration was determined using a Beckman Z2 Coulter Counter (Beckman Coulter, Mississauga, ON). For IVIg or anti-CD44-mediated treatment of thrombocytopenia, mice were injected i.v. with 50 µg KM114 antibody in 200 µL PBS, pH 7.2, or i.p with 50 mg (~2 g/kg) IVIg 30 min prior to the administration of anti-platelet antibody.

### Effect of LPS on IVIg and anti-CD44 amelioration of anti-platelet antibody-mediated thrombocytopenia

To test if LPS would inhibit IVIg or KM114 mediated amelioration of antibody-mediated thrombocytopenia, mice were injected with 1 µg LPS i.p. in 200 µL PBS, pH 7.2, followed 1 hr later by i.p. injection of 50 mg IVIg or i.v. injection of 50 µg KM114 antibody i.v., and 30 min later by i.p. injection of 2 µg MWReg30.

### LPS-mediated thrombocytopenia

To determine if IVIg or KM114 could ameliorate LPS-induced thrombocytopenia, mice were injected with 1 µg LPS i.p., followed 1 hr later by 50 mg IVIg i.p. or 50 µg KM114 antibody i.v.; alternatively, mice were pretreated with IVIg or KM114, followed 1hr or 24 hr later by LPS injection.

### Statistics

Statistics were calculated using a one-way ANOVA with a Tukey post test and data are presented as mean ± SEM.

## Results

### Acute exposure to the TLR4 agonist LPS does not interfere with IVIg or KM114 mediated amelioration oif murine ITP

**Figure 1:**
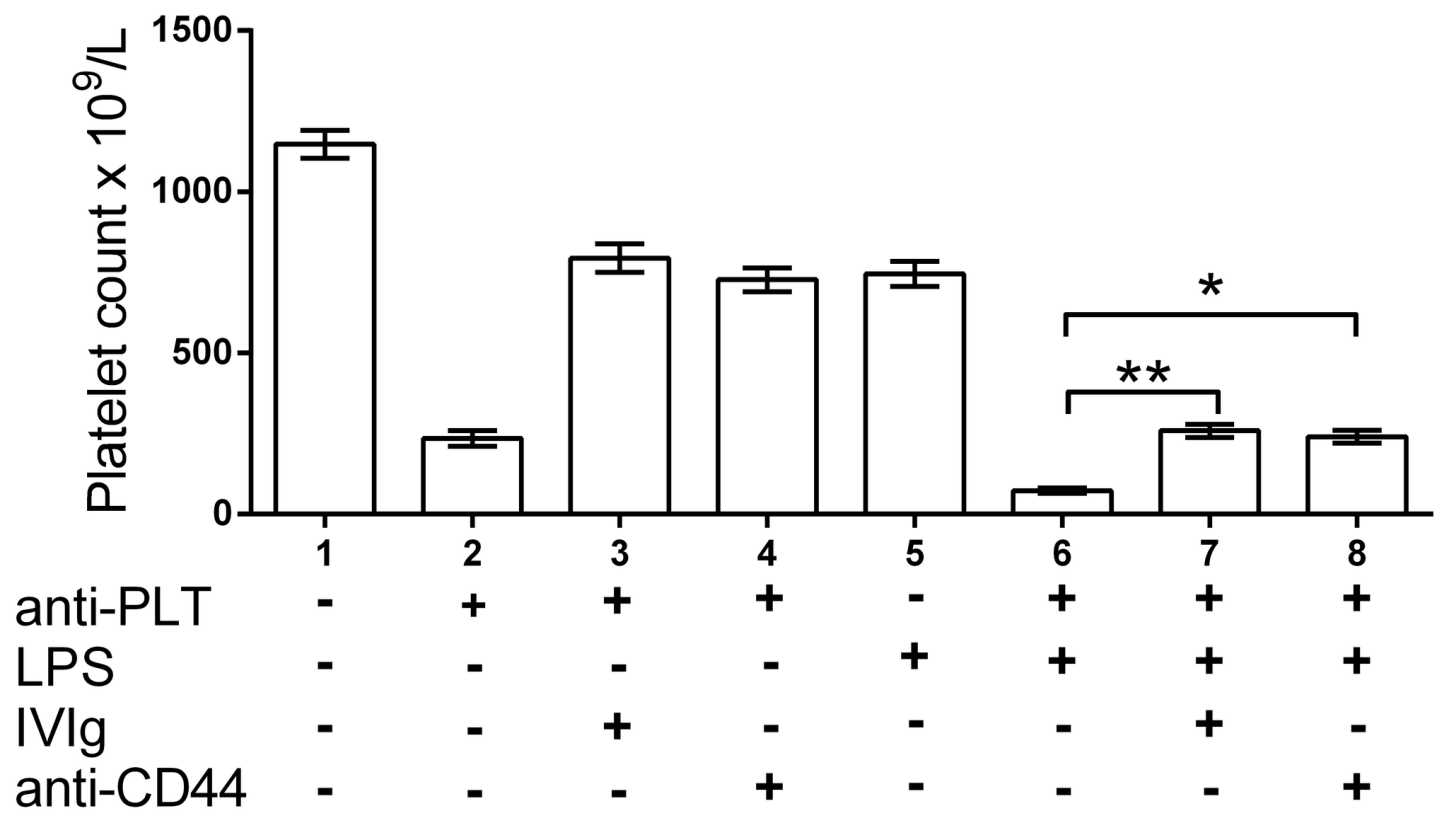
Acute pretreatment of mice with the TLR4 agonist LPS does not affect the ability of IVIg or the CD44 antibody KM114 to ameliorate antibody-mediated thrombocytopenia. CD1 mice were pretreated with nothing (columns 1-4) or with 1 µg LPS in 200 µL PBS (columns 5-8). One hr later, mice were injected with nothing, 50 mg IVIg (columns 3, 7) or 50 µg of the anti-CD44 antibody KM114 in 200 µL PBS (columns 4, 8). Thirty min later, mice in columns 2-4 and 6-8 received 2 µg MWReg30 in 200 µL PBS. Twenty-four hr later, all mice were bled for platelet enumeration. The x-axis denotes mouse treatment groups; the y-axis denotes mouse platelet count. n=6 mice per group from 3 separate experiments of 2 mice each group. Data are presented as mean ± SEM. *P<0.05; **P<0.01.

To investigate the potential role TLRs might play in the mechanism of action of IVIg or KM114, we utilized the murine passive ITP model in which both these therapeutics successfully inhibit thrombocytopenia [32,36]. We initially questioned if the TLR4 agonist LPS would affect the therapeutic abilities of IVIg or KM114. Mice injected with anti-platelet antibody demonstrated significant thrombocytopenia compared to unmanipulated mice (Figure 1, column 2 vs column 1). Pretreatment of mice with either IVIg (Figure 1, column 3) or KM114 (Figure 1, column 4) protected mice against thrombocytopenia. Mice injected with LPS alone (25 hr prior) exhibited a mild thrombocytopenia (Figure 1, column 5), whereas mice injected with LPS and anti-platelet antibody demonstrated a more severe thrombocytopenia (Figure 1, column 6 vs column 2). Exposure of mice to LPS for 1 hr did not affect the subsequent ability of IVIg or KM114 to inhibit anti-platelet antibody mediated thrombocytopenia (Figure 1, columns 7 and 8, respectively).

**Figure 2:**
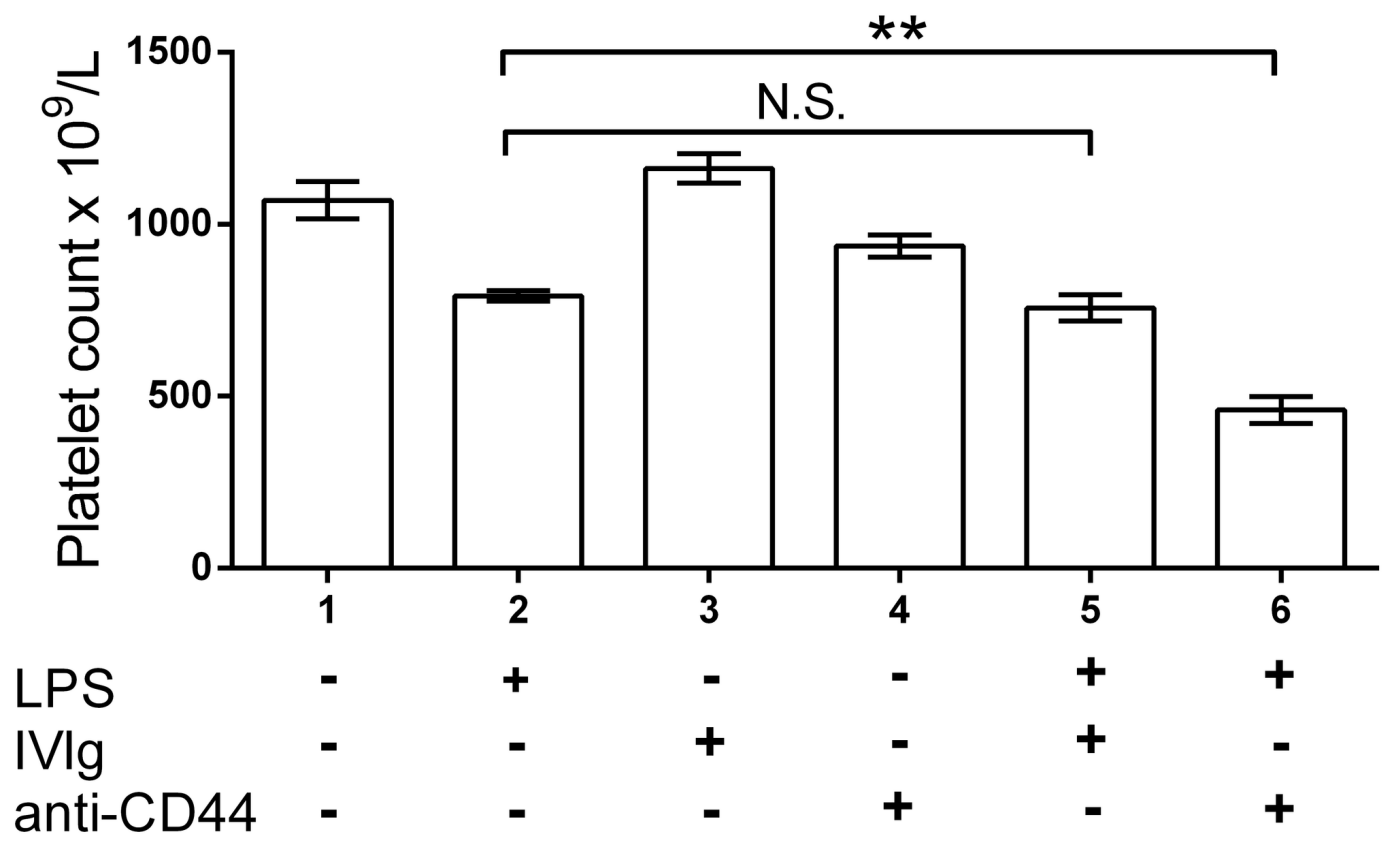
Neither IVIg nor the CD44 antibody KM114 ameliorate acute LPS-mediated thrombocytopenia. CD1 mice were pretreated with nothing (columns 1, 3, 4) or 1 µg LPS in 200 µL PBS (columns 2, 5, 6). One hr later, mice were injected with 50 mg IVIg (columns 3, 5) or 50 µg KM114 in 200 µL PBS (columns 4, 6). Twenty-four hr later, all mice were bled for platelet enumeration. The x-axis denotes mouse treatment groups; the y-axis denotes mouse platelet count. n=6 mice per group from 3 separate experiments of 2 mice each group. Data are presented as mean ± SEM. **P<0.01. N.S. no significant difference.

While IVIg and KM114 did protect against anti-platelet antibody mediated thrombocytopenia in the presence of LPS, the platelet counts did not fully recover (Figure 1 columns 7 vs 3 for IVIg; columns 8 vs 4 for KM114). We questioned whether this observation was due to the inability of IVIg or KM114 to ameliorate LPS induced thrombocytopenia or whether LPS + anti-platelet antibody induced thrombocytopenia was partially resistant to treatment. Mice injected with LPS alone demonstrated mild thrombocytopenia (Figure 2, column 2 vs column 1), while IVIg alone did not affect platelet counts (Figure 2, column 3), and mice receiving KM114 alone displayed some thrombocytopenia (Figure 2, column 4). Mice pretreated with IVIg demonstrated no protection from LPS-induced thrombocytopenia (Figure 2, column 5). Mice pretreated with KM114 also demonstrated no protection from LPS-induced thrombocytopenia but rather showed significantly more thrombocytopenia than mice treated with LPS (Figure 2, column 6 vs column 2) or KM114 alone (Figure 2, column 6 vs column 4).

**Figure 3:**
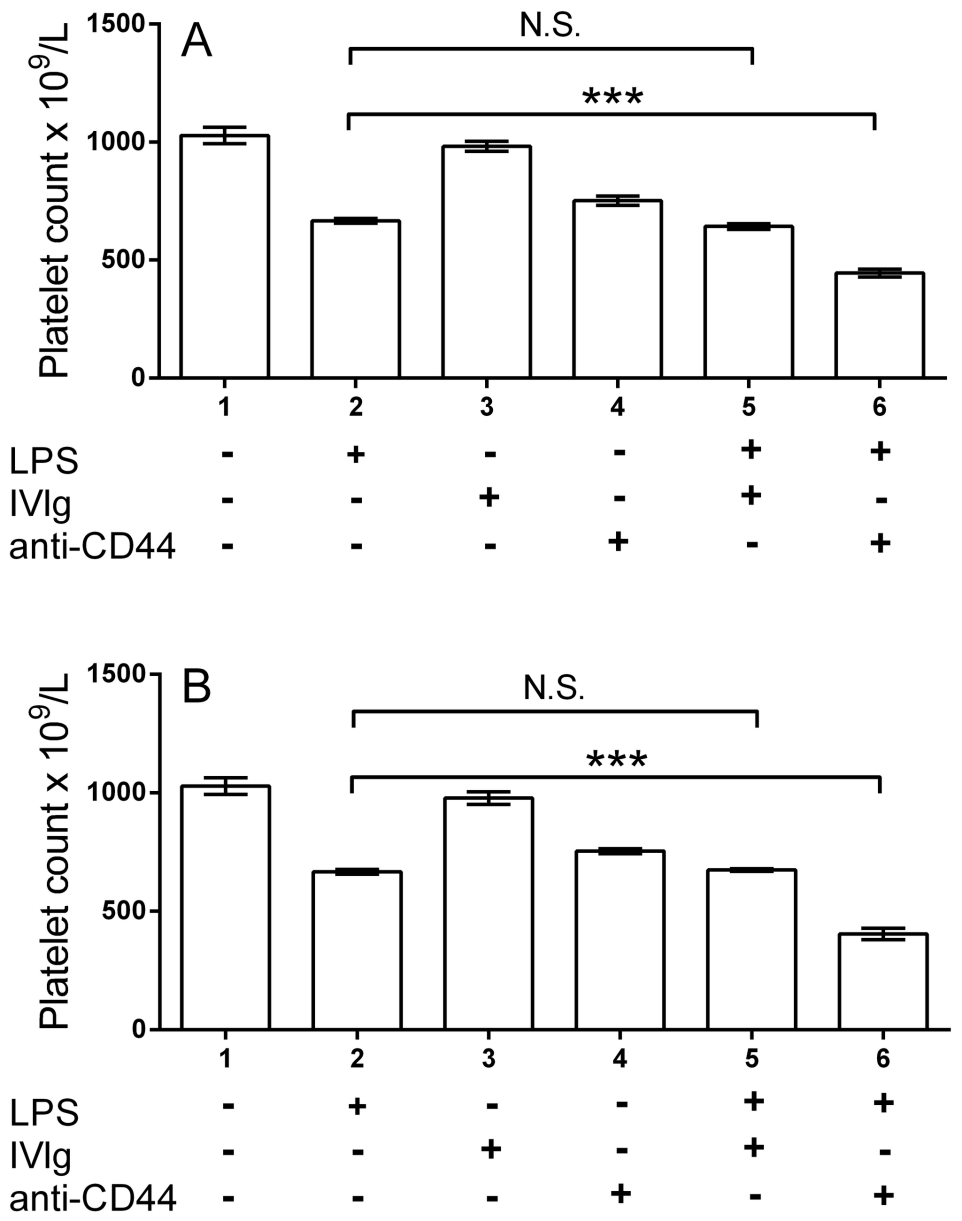
Pretreatment of mice with IVIg or the CD44 antibody KM114 does not prevent the induction of LPS-mediated thrombocytopenia. (A) CD1 mice were pretreated with nothing, 50 mg IVIg (columns 3, 5) or 50 µg KM114 in 200 µL PBS (columns 4, 6). One hr later, mice in columns 2, 5 and 6 received 1 µg LPS in 200 µL PBS. Twenty-four hr later, all mice were bled for platelet enumeration. The x-axis denotes mouse treatment groups; the y-axis denotes mouse platelet count. n=6 mice per group from 3 separate experiments of 2 mice each group. Data are presented as mean ± SEM. (B) Mice were treated as in (A) except LPS was given 24 hr post IVIg or KM114 treatment. ***P<0.001. N.S. no significant difference.

To determine if IVIg or KM114 could inhibit the induction of LPS induced thrombocytopenia, we treated mice with these therapeutics 1 hr (Figure 3A) vs 24 hr (Figure 3B) prior to LPS injection. Neither IVIg nor KM114 had any inhibitory effect on the induction of LPS induced thrombocytopenia. Mice receiving KM114 and LPS again exhibited enhanced thrombocytopenia.

### IVIg ameliorates murine ITP in mice with defective TLR4 signaling

**Figure 4:**
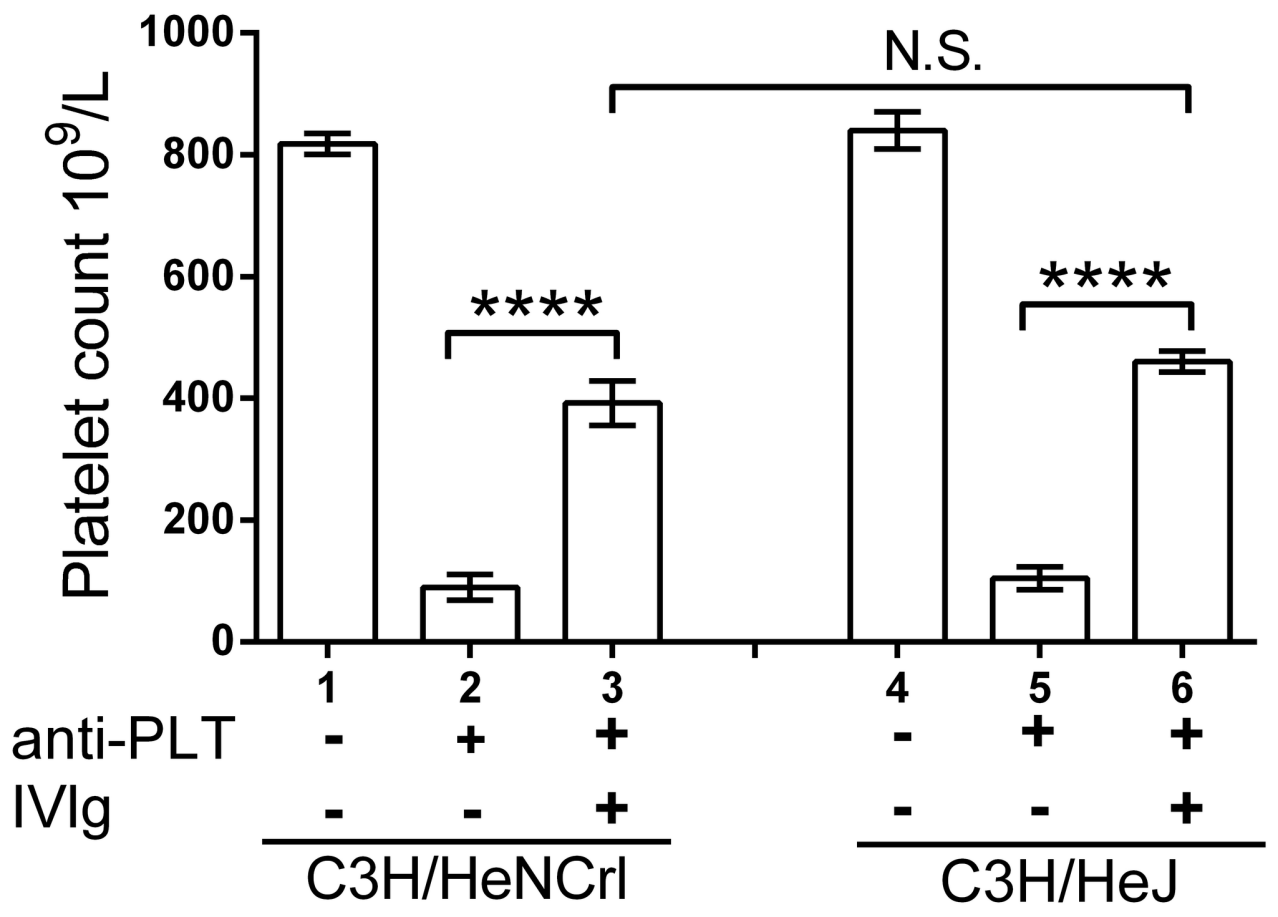
Mice deficient in TLR4 function are protected from immune thrombocytopenia by IVIg. Control C3H/HeNCrl mice and TLR4 deficient C3H/HeJ mice were left untreated (columns 1, 2 and 4, 5) or therapeutically treated with 50 mg IVIg (columns 3, 6). Thirty min later, all mice except ‘Nil’ (columns 1, 4) received 2 µg MWReg30, in 200 µL PBS. Twenty-four hr later, all mice were bled for platelet enumeration. The x-axis denotes mouse strain and treatment groups; the y-axis denotes mouse platelet count. n=6 mice per group from 3 separate experiments of 2 mice each group. Data are presented as mean ± SEM. ****P<0.0001. N.S. no significant difference.

C3H/HeJ mice have a missense point mutation within the cytoplasmic portion of TLR4 and do not express functionally active TLR4 [37–39]. Injection of C3H/HeJ mice or control C3H/HeNCrl mice (express functional TLR4) with anti-platelet antibody resulted in severe thrombocytopenia (Figure 4, columns 2 vs 5) with no discernable difference in platelet counts between the 2 groups. Pretreatment of C3H/HeNCrl mice or C3H/HeJ mice with IVIg protected against thrombocytopenia (Figure 4, columns 3 vs 2 and columns 6 vs 5).

### The presence of Myd88 is not required for IVIg or KM114 mediated amelioration of murine ITP

**Figure 5:**
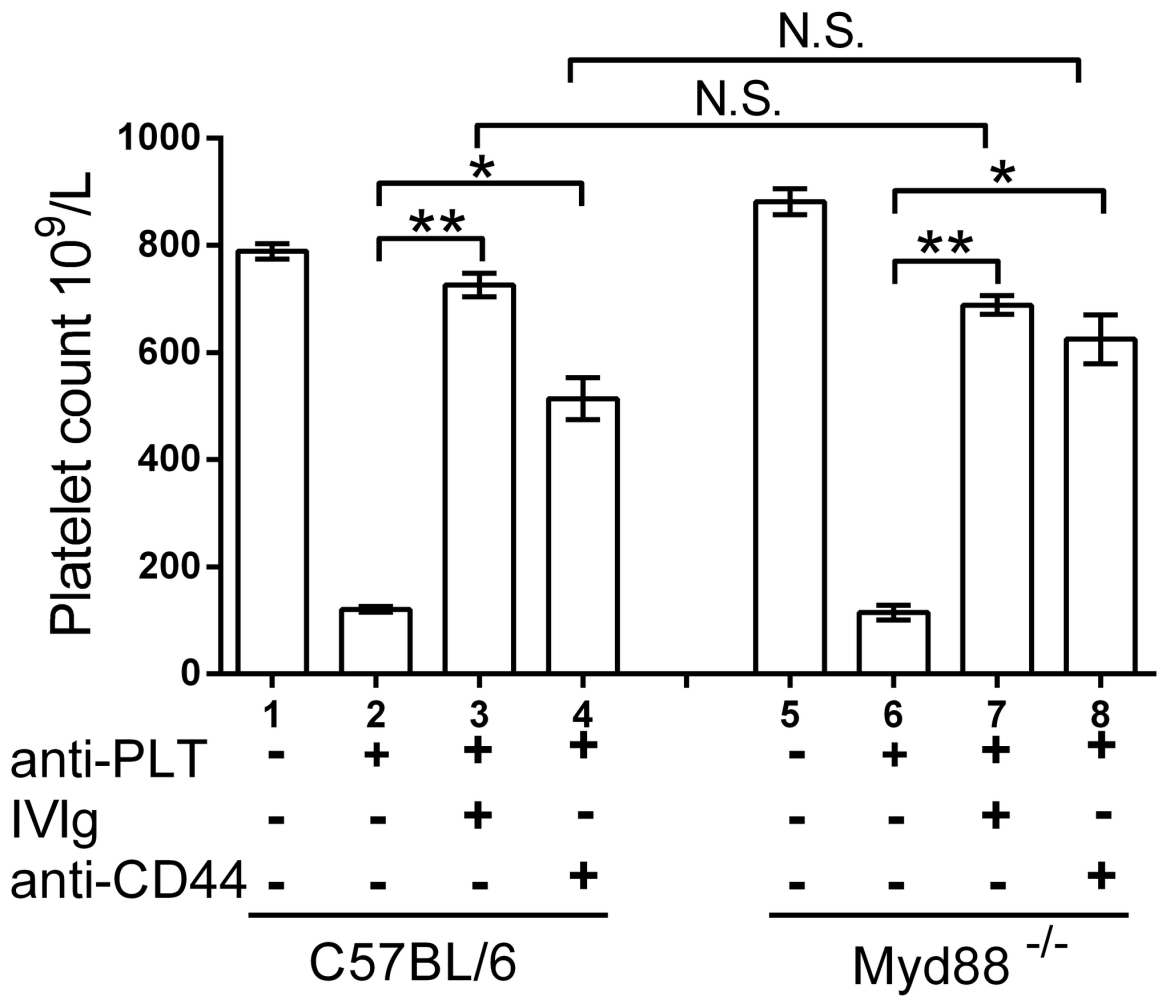
Mice lacking the Toll-Like Receptor adaptor protein Myd88 are protected from immune thrombocytopenia by both IVIg and the CD44 antibody KM114. Control C57BL/6 mice and Myd88 deficient mice (B6.129P2(SJL)-*Myd88* ^*tm1.1Defr*^/J) were left untreated (columns 1, 2 and 5, 6) or therapeutically treated with 50 mg IVIg (columns 3, 7), or 50 µg of the anti-CD44 antibody KM114 in 200 µL PBS (columns 4, 8). Thirty min later, all mice except ‘Nil’ (columns 1, 5) received 2 µg MWReg30 in 200 µL PBS. Twenty-four hr later, all mice were bled for platelet enumeration. The x-axis denotes mouse strain and treatment groups; the y-axis denotes mouse platelet count. n=4 mice per group from 2 separate experiments of 2 mice each group. Data are presented as mean ± SEM. *P<0.05; **P<0.01. N.S. no significant difference.

To discern whether IVIg might require the Myd88 signaling pathway in the amelioration of ITP, Myd88 deficient mice were used (Figure 5). Control C57BL/6 mice (Figure 5, column 2) or Myd88 deficient mice (Figure 5, column 6) injected with anti-platelet antibody both displayed thrombocytopenia with no significant difference in platelet counts between the 2 groups, consistant with phagocytosis of antibody opsonized platelets occurring in the absence of Myd88. Pretreatment of Myd88 deficient mice with IVIg protected against thrombocytopenia (Figure 5, column 7).

To investigate if the CD44 antibody KM114 required Myd88 expression for the amelioration of murine ITP, we also treated Myd88 deficient and control mice with KM114 in the ITP model and found that both control mice (Figure 5, column 4) and Myd88 deficient mice (Figure 5, column 8) were protected from thrombocytopenia, with no significant difference in platelet counts between the 2 groups.

## Discussion

There are multiple theories as to the mechanism of action of IVIg in the treatment of ITP and other autoimmune diseases. Although many cell types have been implicated as targets in IVIg amelioration of autoimmunity, including DC, macrophages, B cells and T cells, the complete pathway(s) by which IVIg functions remains unclear (reviewed in 40,41). We [9–11] and others [8,12–17] have shown that DC can play a key role as initiators of IVIg effects. Using a murine model of arthritis, it has been shown that targeting the DC C-type lectin (DC-SIGN) on DC or bone marrow derived macrophages with IVIg can induce IL-4 production by basophils, leading to increased levels of the inhibitory receptor FcγRIIB on macrophages, which the group suggested may provide a mechanism for maintaining immune homeostasis [12]. However, we have results inconsistent with this mechanism as IVIg functions effectively in mice genetically deficient in IL-4 or the common cytokine receptor γ chain (required for signal transduction through the receptors for IL-2, -4, -7, -9, -15, and -21) [42]. It has also been demonstrated that IVIg can decrease B cell activation and proliferation [43]. In addition, Th_17_ cells, which have been implicated in autoimmune pathogenesis (reviewed in 44), are negatively affected by IVIg in terms of differentiation and amplification [45,46]. Thus IVIg, in addition to its acute effects, may modulate both innate and acquired immunity, potentially resulting in more long term protection.

In addition to IVIg, we have also demonstrated that antibodies to some select cellular antigens [7] including the CD44 antigen [32,36] can ameliorate thrombocytopenia in the murine ITP model. Similar to IVIg in treating murine ITP, we have found that CD44 antibodies function independent of FcRn expression [36] and do not function in FcγRIIB deficient mice on the B6; 129S background [32]; dissimilar to IVIg [6], CD44 antibodies do not require the Fc portion to function therapeutically in murine ITP [32]. Thus IVIg and therapeutic CD44 antibodies show some mechanistic similarities in the treatment of murine ITP.

Toll-like receptors (TLRs) are a class of pattern-recognition receptors that play a key role in activation of the innate immune system (reviewed in 18). However, excessive TLR activation can disrupt immune homeostasis, which may be responsible for the development of inflammation and autoimmune disease (reviewed in 19). It has also been suggested that inappropriate activation of TLRs by endogenous or exogenous ligands, or defective TLR-mediated regulation of regulatory T cells, could provide a possible link between TLRs and autoimmunity (reviewed in 47). Relevant to ITP, it has been shown that *in vitro* ligation of TLR7 on macrophages from ITP patients increases IL-12 secretion, inducing the differentiation of Th_1_ cells [48], which have been implicated as playing a role in patients with ITP (reviewed in 49). It has also been demonstrated that stimulation of DC from ITP patients through TLR7 induces the production of BlyS, which is crucial for B cell proliferation and differentiation, and that BlyS can enhance anti-platelet antibody production by autoreactive B cells *in vitro* [50].

IVIg can attenuate the production of pro-inflammatory cytokines in human monocytic cells by modulating TLR4-mediated signaling pathways [51]. In addition, IVIg can modulate the maturation of TLR4 primed monocyte derived DC [52]. Immune complexes, which we [24] and others [53,54] have shown can ameliorate murine ITP, can also be used to negatively regulate TLR4-triggered inflammatory responses in macrophages [30]. IVIg requires the expression of the activating Fc receptor FcγRIIIA on dendritic cells to ameliorate murine ITP [10] and it has been demonstrated that the *in vitro* activation of macrophages by immune complexes results in association of TLR4 with FcγRIIIA [28]. IVIg can decrease TLR2 on monocytes in Kawasaki Disease patients, which is enhanced in Kawasaki Disease [55]. TLR9 can play an important role in the pathogenesis of SLE [56,57], and it has been demonstrated that IVIg can suppress B cell TLR9 expression and decrease IL-6 and IL-10 production in ODN-CpG activated B cells [58].

CD44 can directly interact with TLR2 [34] and TLR4 [35], and CD44 can negatively regulate TLR mediated cytokine production [34]. As TLR4 is expressed on both macrophages and DC, we questioned whether IVIg or the CD44 antibody KM114 might utilize the TLR4 pathway in its amelioration of immune thrombocytopenia.

To address this, we initially studied whether or not the TLR4 agonist LPS would interfere with IVIg or KM114 mediated amelioration of murine ITP. LPS administration did not inhibit the ability of IVIg or KM114 to ameliorate antibody induced thrombocytopenia, suggesting that putative ligation of TLR4 with LPS does not affect the therapeutic properties of IVIg or KM114. LPS can also induce thrombocytopenia, potentially through LPS interaction with the TLR4/Myd88 signaling pathway, and platelet aggregation may play a role in this process [59]. Thus we questioned whether IVIg or KM114 could protect mice from LPS induced thrombocytopenia. We found, however, that these therapeutics when given prior or post LPS administration demonstrated no ability to ameliorate LPS induced thrombocytopenia. This suggests that even if IVIg can modulate TLR4 signaling [51], it cannot inhibit LPS mediated thrombocytopenia under the conditions studied. In addition, while CD44 can interact with TLR4 [35], ligating CD44 with the monoclonal antibody KM114 also had no effect on LPS mediated thrombocytopenia.

To further study the potential role TLR4 might play in the amelioration of murine ITP by IVIg, we utilized C3H/HeJ mice, which do not express functionally active TLR4 [37–39]. Administration of anti-platelet antibody to either C3H/HeJ mice or control C3H/HeNCrl mice resulted in the same degree of thrombocytopenia in both strains, suggesting that antibody induced thrombocytopenia does not rely on functional TLR4. IVIg was able to significantly ameliorate thrombocytopenia in both mouse strains, with no significant difference in platelet numbers in C3H/HeJ mice vs C3H/HeNCrl mice, suggesting that IVIg can function in the absence of this TLR4 signaling pathway in murine passive ITP.

All murine TLRs except TLR3 require Myd88 for signaling. As a variety of TLRs, including TLR2, 4, 7 and 9 can engage immune complexes [25–31], and CD44 can interact with TLR2 [34], TLR4 [35] and Myd88 [33], we next questioned whether the ameliorative abilities of IVIg or KM114 were reliant on Myd88 expression. The observation that anti-platelet antibody resulted in thrombocytopenia in Myd88 deficient mice suggests that, similar to TLR4, Myd88 expression is not required for the induction of thrombocytopenia in the murine ITP model. IVIg was able to significantly ameliorate thrombocytopenia in both mouse strains, with no significant difference in platelet numbers in Myd88 deficient mice or control mice, suggesting that these therapeutics do not require Myd88 expression for its amelioration of murine passive ITP.

The data presented herein suggest that TLR4 and Myd88 pathways are not required for IVIg or an anti-CD44 antibody to increase platelet numbers in murine ITP. We therefore conclude that the TLRs evaluated here are unlikely to play a major role in the mechanism of action of these two therapeutics in murine passive ITP. These results provide further insight into the mechanism of action of IVIg and CD44 antibodies. Although TLRs have been implicated in the pathogenesis of autoimmunity, how these findings might relate to human ITP is at present unknown.
